# Exploring health promotion efforts for non-communicable disease prevention and control in Ghana

**DOI:** 10.1371/journal.pgph.0002408

**Published:** 2023-09-25

**Authors:** Mark Fordjour Owusu, Joseph Adu, Benjamin Ansah Dortey, Sebastian Gyamfi, Ebenezer Martin-Yeboah

**Affiliations:** 1 School of Health Sciences, University of Canterbury, Christchurch, New Zealand; 2 Department of Health and Rehabilitation Sciences, Western University, Ontario-Canada; 3 Trauma and Specialist Hospital, Winneba, Ghana; 4 Lawson Health Research Institute, London, Ontario, Canada and Arthur Labatt School of Nursing, Western University, London, Ontario, Canada; 5 Department of Health Information Science, Western University, London, Ontario, Canada; Pai Chai University, REPUBLIC OF KOREA

## Abstract

Noncommunicable diseases (NCDs) are a growing public health challenge in Ghana. Health promotion can provide useful avenues to reduce the incidence of NCDs in the country. We used the Ottawa Framework to assess health promotion efforts for the prevention and control of NCDs in Ghana. Data were collected using key informant interviews and documentary sources. A content analysis approach was adopted for data analysis using Nvivo 11 Software. We found a strong policy framework for NCD prevention in Ghana with the ratification of several international protocols and resolutions and the development of national and specific NCD-related policies. Implementation of these policies, however, remains achallenge due to limited resources and the overconcentration on communicable diseases. Attempts have been made to create a supportive environment through increased access to NCD services but there are serious challenges. Respondents believe the current environment does not support healthy eating and promotes unhealthy use of alcohol. The Community-based Health Planning and Services (CHPS) program engenders community participation in health but has been affected by inadequate resources. Personal skills and education programs on NCDs are erratic and confined to a few municipalities. We also found that NCD services in Ghana continue to be clinical and less preventative. These findings have far-reaching implications for practice and require health planners in Ghana to pay equal attention in terms of budgetary allocations and other resources to both NCDs and communicable diseases.

## Introduction

Non-communicable diseases (NCDs) have been on the agenda of public health experts and governments in recent times. This has been underscored by their inclusion in the Sustainable Development Goals (SDGs) after being omitted from the Millennium Development Goals (MDGs). One of the targets of the SDGs is to reduce premature deaths from NCDs through prevention and treatment and promote mental health and well-being [1]. This is particularly important as currently, NCDs have been implicated in 71% of all global deaths, or 41 million fatalities, with a significant percentage occurring in low and -middle-income countries (LMICs) [2]. Although these alarming statistics should provide additional impetus for African countries to tackle NCDs, this has not been the case. There is a historical reason for this, with health systems in African countries traditionally built for the management of infectious diseases [3]. In Ghana, a sub-Saharan African country, research shows that NCDs have reached epidemic proportions [4–6]. Yet there is a dominant belief that NCDs are not as dangerous as infectious diseases, hence their exclusion “from policy analysis and decision making” [7 p.380]. This has negatively impacted efforts aimed at preventing and controlling NCDs.

Over two decades ago, Sinhall [8] advised health planners not to betray the Ottawa Charter by tapping into the opportunities it offered for the prevention of NCDs. Kumar and Preetha [9 p.5] captured this succinctly when they averred that health promotion can “help tackle the unfinished agenda of communicable diseases, newly emerging and re-emerging diseases as well as the unprecedented rise of chronic NCDs”. Health promotion interventions can improve health and wellbeing by encouraging the adoption of healthy lifestyles, preventing disease and injuries, helping to create a suitable environment that supports health, reducing harm, and encouraging advocacy and research. However, due to limited research, it is not clear how health promotion is contributing to the prevention of NCDs in Ghana. Although NCD research is gathering traction in recent years in Ghana, there is still a limited emphasis on the role of health promotion in prevention and control efforts. Health promotion activities continue to centre on infectious diseases such as malaria and diarrhoeal conditions, and less on NCDs [10]. It is, therefore, important to evaluate the role of health promotion in the prevention and control of NCDs in Ghana. This will not only provide additional impetus to the NCD fight in Ghana but will help inform decisions, policies and interventions for the achievement of the SDGs. Our main objective here was, therefore, to assess the extent to which health promotion has influenced the prevention and control of NCDs in Ghana.

### The Ottawa Charter

We adopted the Ottawa Charter Framework of Health Promotion to underpin our study. The Ottawa Charter was the first attempt to foreground health promotion as an integral activity of functioning health systems following the international conference organized by the WHO, the Canadian Government, and the Canadian Public Health Association [11]. This framework enjoins policymakers and health managers to focus on five action areas in their efforts to promote health. These include the development of appropriate policies, creating a supportive environment that promotes health, strengthening community action and participation in health, developing personal skills to improve health, and reorienting health services. The five action areas have been built on the premise that promoting health includes bringing health onto the agenda of policymakers across sectors with the view to encouraging them to understand the consequences of their decisions on health as well as paying attention to the natural and built environment to improve conditions that support healthy living. Also, strengthening community action through empowerment and self-determination in the identification of priority areas and decision-making, encouraging personal and social development through the provision of information and education to increase awareness of health issues, and focusing on the health needs of the ‘whole person’ with emphasis on prevention through the inclusion of sectors other than health are important aspects of this conceptualization. As an internationally accepted framework, the Ottawa Charter was adopted for the current study as it provides a clear structure for assessing NCD-related health promotion activities in Ghana.

## Methods

We explored health promotion efforts for NCDs at the national level in Ghana. Here, we present a brief vignette of the methods from a comprehensive study that was reported elsewhere [12]. For deeper understanding of health promotion for NCDs in Ghana, a qualitative approach involving key informant interviews with participants at the national level and documentary sources was used. Overall, 15 key informants were purposively selected from policymakers (Ministry of Health-MOH), policy implementers (Ghana Health Service-GHS), payers (National Health Insurance Authority-NHIA) as well as advocacy (Ghana Medical Association-GMA) and patient organizations (Ghana Diabetes Association-GDA). Key informants were selected based on roles in their respective institutions, with a working experience of two years in current roles required for selection. With the help of the management of selected organizations, participants voluntarily filled out consent forms to be part of the study.

Documentary data were used to augment and validate interview data. Documentary data came in three formats and included policy and program documents from the MOH and other partners such as the WHO, GHS Annual Reports, and miscellaneous sources including the Common Management Arrangement documents and the Holistic Assessment reports of the health sector.

Using a content analysis approach [13], data analysis was underpinned by an adaptation of Gale et al.’s [14] framework and included preparing the data, importing data into Nvivo 11 Software, coding, utilizing a model for analysis, sorting, and interpretation (See Owusu 2019 for more details). Ethical approvals for this study were obtained from the Ghana Health Services’ Ethics Review Committee (# GHS-ERC:15/06/17) **and the University of Canterbury’s Human Ethics Committee (Ref: HEC 2017/49).**

### Findings

Findings have been presented using the Ottawa Framework as this presents a systematic way of understanding health promotional efforts for NCDs in Ghana.

### Building healthy public policies

Our findings show that a robust policy environment has been established in Ghana in response to NCDs. The current NCD policy which was developed in 2022 specifically captures the five action areas of the Ottawa Charter and places health promotion at the heart of primary prevention [15]. Documentary sources revealed three categories of NCD policies: International policies and resolutions, national policies, and specific NCD policies [16]. Table 1 presents a synopsis of the policy framework for the management and prevention of NCDs in Ghana.

**Table 1:** The policy framework for NCD prevention in Ghana.

International Resolutions	National Health Policies	Specific NCD Policies
WHA Request for a Global Strategy for NCD Prevention and Control	Ghana Shared Growth and Development Agenda (GSGDA), 2010–2013	National NCD Policy, 2012; 2022
Reaffirmation of Global Strategy of NCD Prevention and Control	National Health Policy 2007	National Strategy for the Prevention and Control of Noncommunicable Diseases 2012
Transparency in Tobacco Control Process	Health Sector Medium Term Development Plan 2010–2013	Ghana National Nutrition Policy 2014–2017
Development of a Global Strategy on Diet, Physical Activity and Health (DPAS)	Health Promotion Policy 2005	Ghana Tobacco Control Regulations 2016 (L.I. 2247)
Adoption of WHO Framework Convention on Tobacco Control (FCTC)	Child Health Policy 2007–2015	National Alcohol Policy, 2016
Endorsement of DPAS	Regenerative Health and Nutrition Programme Strategic Plan 2007–2011	
Health Promotion and Healthy Lifestyles	Disease Control Strategy 2010–2014	
Public Health Problems caused by Harmful Use of Alcohol		
Prevention and Control of NCDs: Implementation of the Global Strategy. Call to Prepare an Action Plan		
Endorsement of a six-year Global Action Plan 2008–2013		
Strategy for the African Region on NCDs, WHO AFRO 2000		
WHO Framework Convention on Tobacco Control, 2003		
Global Strategy for Diet, Health and Physical Activity, 2004		
Action Plan for the Global Strategy for the Prevention and Control of NCDs, 2008		

When asked about the policy environment for NCDs, key informants stated that Ghana has developed the requisite policies to control NCDs. However, respondents were unanimous about the challenges of implementation. A key informant commented;

*As part of our program, we know NCDs are a national problem. We have the policies, the strategies, and the action plans. We have made a national NCD policy and an NCD Strategy. The unfortunate thing is that the resources to implement the policies is what is lacking [Policy implementer 2]*.

### Creating a supportive environment for noncommunicable disease prevention

On whether a supportive environment has been created for NCD prevention, the findings were mixed. Some key informants stated that policymakers have been conscious of the role of health promotion in the NCD fight for a while. A key informant explained;

*The 2007 National Health Policy was anchored on healthy lifestyle and health promotion*. *What it means is that the MOH was thinking about NCDs even at that time…*.*Also*, *around that time the then Minster of Health*, *Major Rtd Courage Quashigah was a strong advocate of health promotion and healthy lifestyle…*.*[Policymaker 2*].

Another key informant added that the structure of the delivery of NCD services shows that a supportive environment has been created.

*Right from the community level*, *we have Community-based Health Planning and Services (CHPS) which are even in hard-to-reach areas where you have basic equipment to test BP and other services*, *screening etc*. *From that basic level of NCD service delivery*, *we have health centres*, *clinics*, *and hospitals*, *both district and regional*. *…*..*The government has also introduced the National Health Insurance Scheme [NHIS] to improve financial access to NCD services [Policy implementer 3*].

Documentary analysis revealed some of the measures undertaken in the past to create a supportive environment for the prevention and control of NCDs. These include advocacy, research, and risk factor control measures presented in Table 2 below.

**Table 2:** Measures aimed at creating a supportive environment for NCD prevention in Ghana.

Intervention	Documentary source
Celebration of international days	GHS Annual Report, 2011
Fiscal levers for healthy food and drinks	National NCD Policy, 2022
Advocacy for physical education sessions in schools	National NCD Policy, 2012; 2022
Raising revenue for advocacy organizations by increasing taxes on tobacco and alcohol products (sin taxes)	National NCD Policy, 2012
Advocacy on NCD related research	National NCD Policy, 2012
Advocacy for healthy eating in curricula of schools	National NCD Strategy, 2012
Advocacy for national stakeholder support on nutrition	National Nutrition Policy, 2013

Some informants, however, thought a supportive environment has not been created. Three respondents spoke about a failure to control risk factors, adding that the inability to regulate the mass media and trade makes it difficult to fight NCDs. A key informant from an advocacy organization commented as follows;

*In actual fact*, *we are rather making it worse*. *Let me give you one example*, *when you listen to all the FM stations in Ghana and you watch prime-time news on TV*, *what do you see*? *Promotion of bitters*. *The whole place is flooded with Adonko Bitters [a local alcoholic bitters] adverts……Also*, *we all want to eat fast food in restaurants… NCDs will not go down*. *So*, *the current psyche of the nation does not help in the fight against NCDs [Advocacy organization participant 1*].

Some key informants also thought that a supportive environment will be created when equal attention is given to both NCDs and communicable diseases, adding that for this to happen, there should be more funding for NCDs. One commented;

*There are certain conditions or diseases that are the focus of the world*. *These include malaria*, *HIV/AIDS*, *TB…*…*So*, *there is a focus on these conditions because of international attention*, *the reason being that they are communicable diseases that can spread among populations very rapidly and thus have serious public health implications*. *But when you come to NCDs*, *I don’t know whether it’s this SDGs that we are going to focus on because I have not seen any funding set aside to combat these conditions [Policy implementer 1*].

### Strengthening community action

On community action for health, policymakers spoke about the CHPS as a community-based program that is meant to empower local people to own initiatives and make key decisions about their own health. It was added that important community institutions such as volunteers and Local Health Committees have been created to engender community participation in NCD efforts. However, respondents added that inadequate resources pose a big challenge in some districts. One informant explained this in some detail;

*We have brought services to the doorsteps of communities through the CHPS……We have even gone further to use community volunteers*. *The CHPS means that the health worker should not sit in one place and wait for people to come to them*. *CHPS is a paradigm shift*. *Community volunteering is a kind of orthodox dovetailing into the community…But in order to dovetail well*, *you need the help of the community*. *They need to own it and then you are there to facilitate the whole thing…*.. *But we still have motorbike issues and the volunteers do not even have bicycles to deliver some of the messages and commodities [Policy implementer 4*].

Resource issues reverberated across participants’ responses. The main issue here was that more resources are required to stimulate effective community action in terms of health promotion for NCDs. A key informant explained;

*You submit your budget and the funds do not come*. *So*, *you are looking up to donors and they are not interested especially in NCDs*. *So*, *if you talk to some of the staff at the local level*, *they will talk with pain*. *It’s like knowing what to do and yet your hands are tied [Policy implementer 2*].

### Developing personal skills

Analysing responses on the development of personal skills showed that there are NCD clinics (education programs) in hospitals where patients are educated about how to manage NCDs. However, our study found that this does not happen in all hospitals in the country. Some policymakers generally believe that personal skills rest with individuals in lifestyle choices and behaviour. A key informant commented;

*The problem is that our thinking has been that these conditions do not have immediate public health implications*, *they are a bit not appreciated as communicable diseases*. *For example*, *if I have hypertension*, *I don’t give it to you*. *It’s my own headache and a lot has to do with lifestyle*. *So*, *even donors are thinking that we should be responsible and live right [Policy implementer 1*].

This notwithstanding, documentary sources revealed that the MOH and the GHS have partnered with international bodies for NCD health promotion purposes. The Buddy Doctor Initiative [BDI] and the Base of the Pyramid [BoP] project represent a collaborative effort involving the MOH, Novo Nordisk Pharma and the Danish Government. These initiatives are meant to empower doctors and nurses to support diabetic patients to improve self-management through increased education and care [17]. However, these are limited to hospitals in a few municipalities. Personal skills programs are erratic and organised by patient organizations, philanthropic bodies, advocacy groups and district health authorities when resources permit. A respondent from a patient organization commented;

*Every quarter we educate and screen one community…*.*Last quarter we had it in September at Weija and Oblogo and we had a lot of cases*. *This year due to financial challenges*, *we could not have it in the first quarter*. *We had it in the second quarter*, *June to be specific at Aplaku and Mallam [Patient organization participant 1*].

### Reorienting health services

Our findings on re-orienting health services for NCDs show that the Ghanaian health system remains tilted towards clinical management, with little emphasis on prevention. A key informant stated;

*You see*, *I think a lot of the funding is given to clinical management*… . *that’s what people are interested in because that’s where you buy drugs and then they see that you are doing something and not much about prevention*. *So*, *it’s like wait*, *when you get sick you come [Policymaker 2*].

Documentary sources support this and indicate that NCD management is predominantly clinical in nature [15, 18]. Respondents stated that implementation effort is concentrated on treatment because successive governments have focused on investing in short-term programs that yield visible results. Consequently, although preventive interventions are part of policies and initiatives, they are hardly implemented.

## Discussion

Non-communicable diseases have become a global public health issue, with regulatory and legislative interventions recommended for their effective prevention and control [19]. Our findings show that Ghana has made considerable progress in her efforts to control NCDs. International protocols and resolutions have been ratified, national policies have been developed, and risk-factor policies are in place. Thus, a robust policy structure has been established in line with the Ottawa Framework. The celebration of national and international NCD awareness days, advocacy for healthy eating and physical education in schools are among the measures contributing to the creation of a supportive environment for the NCD fight. The CHPS program has been used to bring health professionals and communities together to fight NCDs. Initiatives such as the BDI and the BoP are being used to equip individuals with the skills for NCD self-management. Finally, clinical support and treatment are available for NCD patients in Ghana, with the establishment of the NHIS pivotal to accessing health services.

While these represent positive strides in health promotion efforts, our key informants also pointed out the inability to implement NCD policies due to financial constraints, with others stating that a supportive environment for NCDs would only be possible if equal attention were given to NCDs and communicable diseases in terms of funding. Financial and logistical constraints negatively affect the CHPS program, impacting effective community participation for NCDs. Personal skills programs for NCD prevention have been erratic and fragmented due to financial constraints, with the BDI and the BoP only available in a few districts. Limited funding also means that clinical interventions receive higher priority in health promotion for NCDs compared to preventive efforts. Thus, two main related issues permeate health promotion efforts in Ghana according to the findings: limited funding and overconcentration on communicable diseases.

As stated in the current NCD policy of Ghana, the MOH recognises the increasing burden of NCDs [15] and policies have been developed to prevent these conditions. This is because policy development is only the first step in health promotion efforts. Developed policies must be effectively implemented and the inability to put policies into action has been confirmed across Africa [20]. The poor implementation of NCD policies which has consequently affected health promotion efforts has been traced to a number of factors including inadequate funding, poor intersectoral partnerships, poor awareness of the severity of NCDs, and inadequate evidence for policy development and implementation [21]. In two separate studies, Juma et al. [22, 23] found inadequate resources as the main challenge in the development of NCD policies and multisectoral action plans in Ghana and other African countries. Limited funding has also been implicated in poor NCD risk factor control efforts, inhibiting effective health promotion [21, 24].

Our findings here have global implications since resource constraints for NCD health promotion transcend national and regional borders. To understand and appreciate this, one must compare NCD funding to communicable diseases on the global scene. As found in this study, donor funding constitutes a major source of health financing especially in LMICs where NCDs are most prevalent [25]. However, studies in the late 2000s show that compared to NCDs, a large percentage of donor funding went into communicable disease prevention. Ravishankar et al. [26] and Stuckler et al. [27] averred that the WHO itself prioritises health promotion for infectious diseases as 87% of its budget in 2006/2007 was allocated to infectious diseases. Sridhar and Batniji’s [28] analysis of the leading health donors revealed startling disproportionate spending in favour of communicable diseases, with HIV/AIDS receiving USD1030 per annual mortality to USD3 for NCDs. Given that NCDs were not captured in the MDGs, these figures were probably not alarming at the time as NCDs had not yet gathered global traction. However, after the 2011 UN Summit and the subsequent inclusion of NCDs in the SDGs in 2015, one would have expected a sharp increase in international funding for NCDs. However, this has not been the case as NCD funding has remained stagnant. In 2017, it was reported that the share of development assistance related to NCDs has remained between 1–2% since 2000 [29]. Jailobaeva et al. [30] reported that although the US and UK governments remain the largest contributors to NCD funding, contributions to bilateral portfolios were only 0.48% and 1.66% respectively, with LMICs depending on philanthropic organizations for financial support. These trends confirm our findings that for effective health promotion for NCDs, equal attention should be given in terms of funding to NCDs and communicable diseases.

Poor global NCD funding subsequently affect national health promotion efforts. In Ghana, the CHPS programme which represents health service response to NCDs and other conditions at the community level has been constricted by inadequate funding [31] while the same can be said of other African countries [32]. Long-term policies in Ghana before the 2000s excluded NCDs [33]. However, the NHIS was introduced in 2004 as the main source of funding for all conditions including NCDs. The scheme has improved access to common NCD services and medications in communities. However, not all services and medicines are covered under the scheme [34]. For example, some cardiac investigations such as angiography are not covered and medications including Candesartan must be paid for by the service user since this is not on the medicines list of the Scheme [35]. With the Scheme struggling with cost-containment [36, 37], poor clients find it difficult to pay for such services and medications out-of-pocket. In one study, it was found that the NHIS had a ‘negative’ impact on the treatment of hypertension because of the length of time it took for the NHIA to reimburse health providers under the scheme [38]. This means that providers are unable to maintain stock levels and usually direct patients to go outside and buy medications that are even covered under the scheme. Thus, although health insurance has improved access to NCD services, it has not solved all the problems associated with financial access.

Although the CHPS strategy has been successful in many communities, more cost-effective community based NCD programs are needed to augment current approaches as these have been known to be particularly successful. The Family Education Diabetes Series (FEDS) project [39] showed how building trust through effective linkages among community leaders, providers and clinical researchers could engender improved diabetes care at the community level. In Pakistan, the Control of Blood Pressure and Risk Attenuation (COBRA) study showed that improved training for community health education workers and physicians coupled with household screening in community clusters could improve NCD outcomes [40]. In Nigeria, Adeyemo et al. [41] found that community-based nurse-led hypertension programs improved medication adherence and saved cost. It seems that rather than using the CHPS as an all-encompassing community health strategy, specific cost-effective community led NCD programs could be the way forward. Ghana is gradually gravitating towards this approach in addition to the CHPS. As was found in this study, the BDI and the BoP Projects are examples of such NCD programs being undertaken in some communities to address NCDs. Another community-based NCD program being implemented in Ghana is the technologically driven Community-based Hypertension Improvement Program (ComHIP) which employs community-driven task shifting methods to control hypertension. While the program’s cost-effectiveness has been questioned compared to standardised hypertension care in Ghana [42], its effectiveness in improving NCD outcomes has been confirmed [43]. Like the other community-based NCD programs found in this study (BDI and BoP), the ComHIP was a collaboration between the GHS and Novartis Foundation-another example of Ghana’s dependency on international organizations in NCD prevention and the need to improve internal resource mobilization for community NCD initiatives.

Ghana has also adopted the World Health Organization’s Package for Essential NCD (WHO-PEN) intervention in some communities. The evidence shows that this intervention has improved NCD outcomes in LMICs including Bhutan [44] and Myanmar [45]. In Ghana’s case, however, human resource and service delivery challenges have been noticed in some communities, with the NHIS struggling to provide all the financial requirements for effective implementation [46]. This notwithstanding, the fact that some of these community-based interventions have been implemented successfully in low resource settings show that if effectively managed, NCD outcomes could improve tremendously. Thus, within the framework of the Ottawa Charter, widening funding sources and increasing budgetary allocation to health can go a long way to support health promotion for NCD activities. This will help implement key policies, support the creation of a conducive environment, and increase the number of community programs and education campaigns needed to engender the lifestyle changes required for NCD prevention.

As shown in this study, Ghana is struggling to control NCD risk factors despite developing tobacco, alcohol, and nutrition policies. This has to do with the socio-cultural evolution of modern Ghanaian societies. The development of fast-food culture in Ghana has been seen as one prime example requiring education on lifestyle modification in terms of healthy eating [47]. This is even more dangerous in Ghana where many people portray fast-food patronage as a display of social status and opulence [48], stressing the importance of prevailing social norms and cultural contexts in determining the effectiveness of health promotion programs and policies. While such ingrained sociocultural issues constipate health promotion efforts, one cannot discount the role of other broader issues in NCD prevention related to globalization and urbanization. The proliferation of multinational fast-food chain outlets in LMICs is a key example. It has been reported, for example, that McDonald’s increased its outlets sevenfold between 1987 and 2002 [49], a phenomenon which has contributed to the ‘export of obesity’ and other metabolic risk factors to LMICs. Thus, health promotion efforts go beyond national NCD campaigns to encompass handling the activities of powerful multinational companies who enter LMICs in the name of investment. While these global behemoths provide employment opportunities and other benefits to local communities, they, nevertheless, make it difficult to reap the full benefits of NCD-related health promotion activities. Some researchers attribute the failure of LMICs to promote health regarding NCDs to the neoliberal idea of choice (the idea that people can make good decisions about their own health), a predominantly western philosophy which is sweeping across LMICs and making it difficult to control NCDs in these countries [50].

Although education and advocacy could play a leading role in changing lifestyles and promoting healthy living, these require sustained interventions rather than the current piecemeal and ad hoc approaches from philanthropic and advocacy groups as well as the occasional celebration of certain days by the MOH and GHS.

Granted, research confirms that non-governmental organizations (NGOs) and advocacy groups could play a key role in NCD prevention by using workshops to train volunteers to enhance personal development and control risk factors [51]. However, this requires greater coordination between these organizations and governments, especially in LMICs where local capacity is inadequate. The problem here is that in Ghana and many LMICs, there are usually no central agencies to coordinate and evaluate the contributions of advocacy groups and NGOs in NCD prevention, a phenomenon which in turn makes it difficult to know how government might support and collaborate with these bodies. According to the findings, programs for personal skills for NCDs are concentrated in a few municipalities in Ghana which usually take place when patients attend hospitals. This has affected awareness levels of NCDs in the general population. In general, awareness levels of NCDs range from 24% to 54% in Ghana [33], with high awareness in urban areas [52]. The poor awareness feeds into the overemphasis on clinical approaches in Ghana, with evidence showing that many people learn about NCDs after being diagnosed [24]. The development of personal skills has been connected to health literacy, the ability to access, understand, and utilize health information to make personal decisions [53]. Since patients with greater health literacy levels are more likely to achieve glycaemic control [54], inadequate educational programs coupled with a largely illiterate population in Ghana contribute to poor personal skills for NCDs.

Contrary to the recommendations of the Ottawa Charter to focus more on preventative interventions, we found little emphasis on prevention in NCD health promotion in Ghana. In addition to documentary evidence, participants stressed the ‘wait until you get sick’ phenomenon. This corresponds to Aikins et al. [55] who explained that Ghana’s orientation and conceptualization of NCDs continues to centre on treatment rather than prevention because these conditions are primarily understood in biomedical terms requiring epidemiological and clinical response, hence, a natural result of Ghana’s understanding and management of NCDs. Aikins and Koram [7] state that policies in many LMICs omit early detection and prevention to focus on treatment and the management of complications of NCDs. This brings to the fore the implementation of the WHO ‘best buy’ interventions which include some cost-effective, evidence-based preventative mechanisms to control NCDs. For example, evidence shows the effectiveness of taxation in controlling tobacco use as a percentage increase in taxes could trigger a double reduction in smoking in LMICs compared to HICs due to the price elasticity there of tobacco products [56]. While this should serve as a useful preventative intervention and incentive in LMICs in terms of tobacco control, they are hardly adopted or underutilised. In Ghana, the 16.1% excise tax on the retail price of cigarettes is far below the WHO benchmark of 70% [57], an indication that tax increases could be a good preventative weapon to reduce the prevalence of smoking even further. It is, therefore, vitally important for LMICs especially those in Africa where NCD deaths are most prevalent, to implement the WHO ‘best buy’ interventions to strengthen preventative efforts. As it stands, the extent of implementation of these interventions are not clearly known in many African countries [58]. Given that financial constraints remain a key challenge in health promotion in Ghana, a focus on the ‘best buys’ will not only improve prevention of NCDs and save lives, but will provide economic gains since these interventions have been known to increase productivity [59].

## Conclusion and implications

We explored health promotion efforts for NCDs in Ghana using the Ottawa Charter framework. The findings of this study provide an extensive appreciation of obstacles leading to the rise in NCD cases in Ghana which have implications for health policy, implementation and research. We found a robust policy framework for NCD prevention in Ghana although implementation of these policies remains a daunting challenge chiefly due to inadequate resources. Some key informants are of the opinion that Ghana has not created the needed supportive environment to control the risk factors associated with NCDs. For instance, the failure of the National Media Commission to regulate the mass media on advertisement of local alcoholic beverages has contributed to the rising incidence of NCDs in Ghana. Also, community efforts and personal skills programs for NCDs remain fragmented due to resource constraints. Thus, although the MOH/GHS and some development agencies have partnered for NCD health promotion purposes, such programs are limited to a few selected municipalities. In general, we found that NCD actions have been more clinical, stressing the need for a shift towards preventative approaches.

Policymakers in Ghana need to strengthen the CHPS program with adequate funding and staff to undertake health promotional activities at the community level on NCDs, highlighting the causes and preventive measures. Also, the government of Ghana and the MOH should take full responsibility for making available budgetary allocations and other resources for NCD programs and activities and avoid the usual overreliance on donor organizations for program funding. The over-concentration on infectious diseases to the neglect of NCDs in Ghana needs revising by the MOH to possibly give equal attention to both sides since individuals with NCDs are sometimes at a higher risk of mortality from infectious diseases as is the case with those with Covid-19. If Ghana is to achieve the SDG goals, then health promotion programs need to be extended to all municipalities to help reduce the incidence of NCDs in the country.
